# Follicular Lymphoma Recurrence Presenting as Unilateral Leg Swelling and Ipsilateral Hydronephrosis

**DOI:** 10.7759/cureus.7199

**Published:** 2020-03-07

**Authors:** Amr Elmoheen, Ali Hassan, Waleed Salem, Khalid Bashir

**Affiliations:** 1 Emergency Medicine, Hamad Medical Corporation, Doha, QAT

**Keywords:** unilateral leg swelling, pocus, follicular lymphoma, hydronephrosis

## Abstract

A 73-year-old man with a history of follicular lymphoma ten years ago with complete remission presented to the emergency room with a large unilateral left-leg swelling as the only symptom. Doppler ultrasonography ruled out deep vein thrombosis. Bedside point-of-care ultrasound (PoCUS) revealed left moderate hydronephrosis. A CT of the abdomen showed a bulky soft tissue mass in the region of the distal left psoas muscle. The mass was generating an extrinsic compression of the left ureter and venous system. The histological study of the lesion established the diagnosis of follicular lymphoma recurrence. The patient subsequently received immunochemotherapeutic treatment in the oncological treatment center.

This case report is intended to promote awareness about a possible clinical correlation between unilateral leg swelling and hydronephrosis with a compressive intra-abdominal mass.

## Introduction

An adequate clinical correlation and the availability of fast and accurate diagnostic methods such as bedside point-of-care ultrasound (PoCUS) are fundamental in the emergency room for the diagnosis of pathologies with an atypical presentation. This report describes an unusual clinical presentation of follicular lymphoma recurrence, which required multidisciplinary management.

Follicular lymphoma is the second most common lymphoma worldwide and represents 30-35% of all non-Hodgkin lymphomas and up to 70% of "low grade" lymphomas [1]. Patients with follicular lymphoma usually have an increased volume of superficial lymph nodes. However, when relapses occur, the extent of the disease increases and lymph nodes can be affected in deep areas (infra-diaphragmatic, retroperitoneum, mesenteric, or iliac regions) and can compress certain structures [2].

The highest incidence is observed in adults in the sixth decade of life, with a male:female ratio of 1:1.7 [3]. The most affected organs are the lymph nodes, spleen, bone marrow, blood, and Waldeyer's ring. Extraganglionic locations can be in the skin, gastrointestinal tract (particularly the duodenum), ocular annexes, breast, and testicles.

Clinically, most patients have an advanced-stage disease, and only 15-20% are in stages I or II at the time of diagnosis. Patients usually have a general preserved state; the development of B symptoms (fever, night sweats, and weight loss) is rare. Patients with follicular lymphoma typically have slightly enlarged superficial lymph nodes, which sometimes go unnoticed. In some patients, the first symptoms manifest themselves insidiously, and they are related to the slow growth of lymph nodes in deep areas, usually in the infra-diaphragmatic, retroperitoneum, mesenteric, or iliac regions. In such cases, patients may complain of nonspecific abdominal symptoms.

## Case presentation

A 73-year-old man presented to the emergency room with a massive unilateral left-leg swelling that had started two months ago. He had been treated for follicular lymphoma ten years ago with a complete course of chemotherapy, which had led to complete remission, His medical history also included laparoscopic cholecystectomy, chronic kidney disease, and parkinsonism for which he was on medication. He denied any history of trauma, fever, rash, vomiting, diarrhea, lower urinary tract symptoms, hematuria, weakness, loss of weight, or night sweats. A thorough physical exam only revealed severe swelling of the whole left lower limb, including the thigh and the leg, when compared with the right lower limb. The patient had mild abdominal distension. The rest of the general and systemic examination was within normal parameters.

Investigations and diagnostic procedures

**Figure 1:**
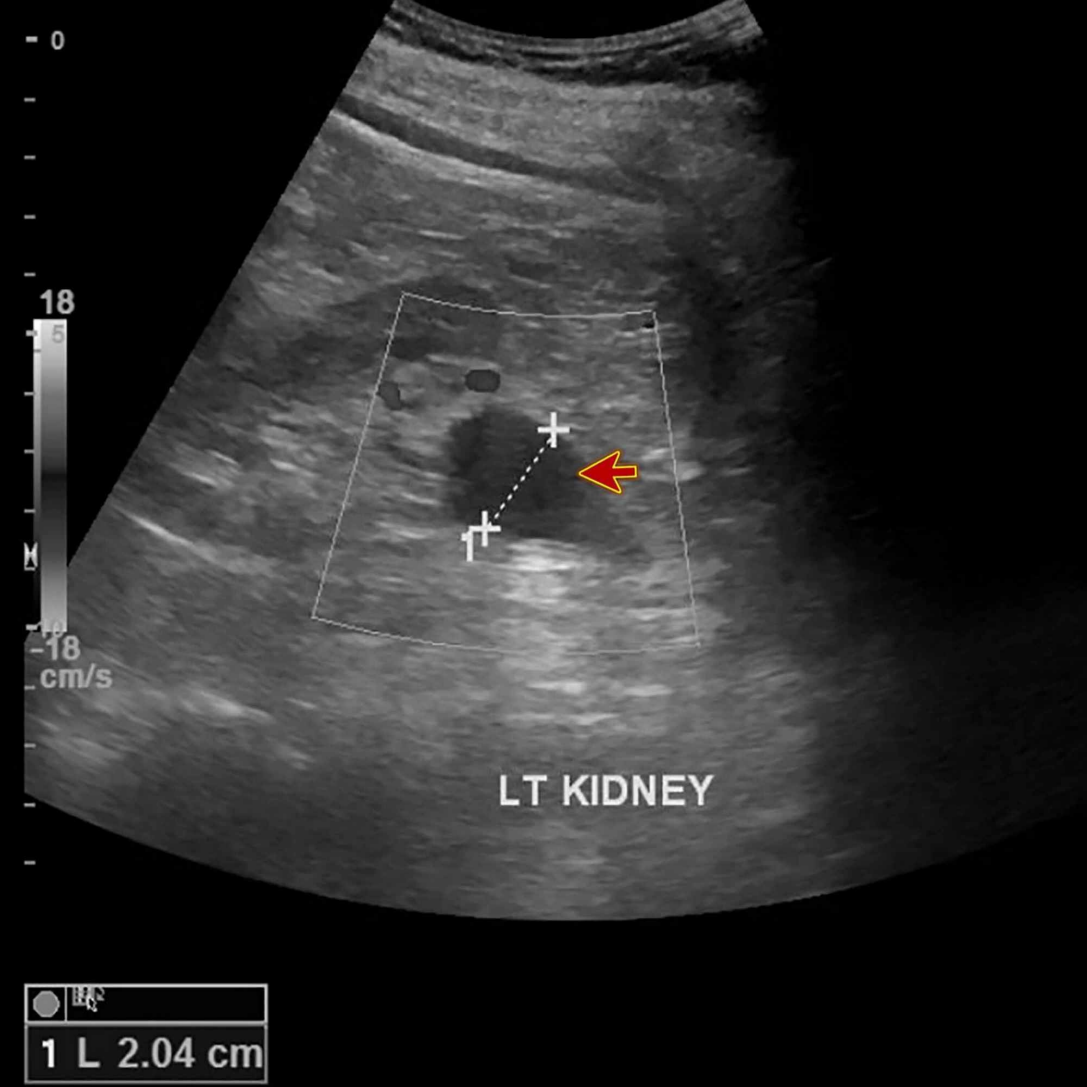
Bedside point-of-care ultrasound The image shows left kidney moderate hydronephrosis (red arrow)

**Figure 2:**
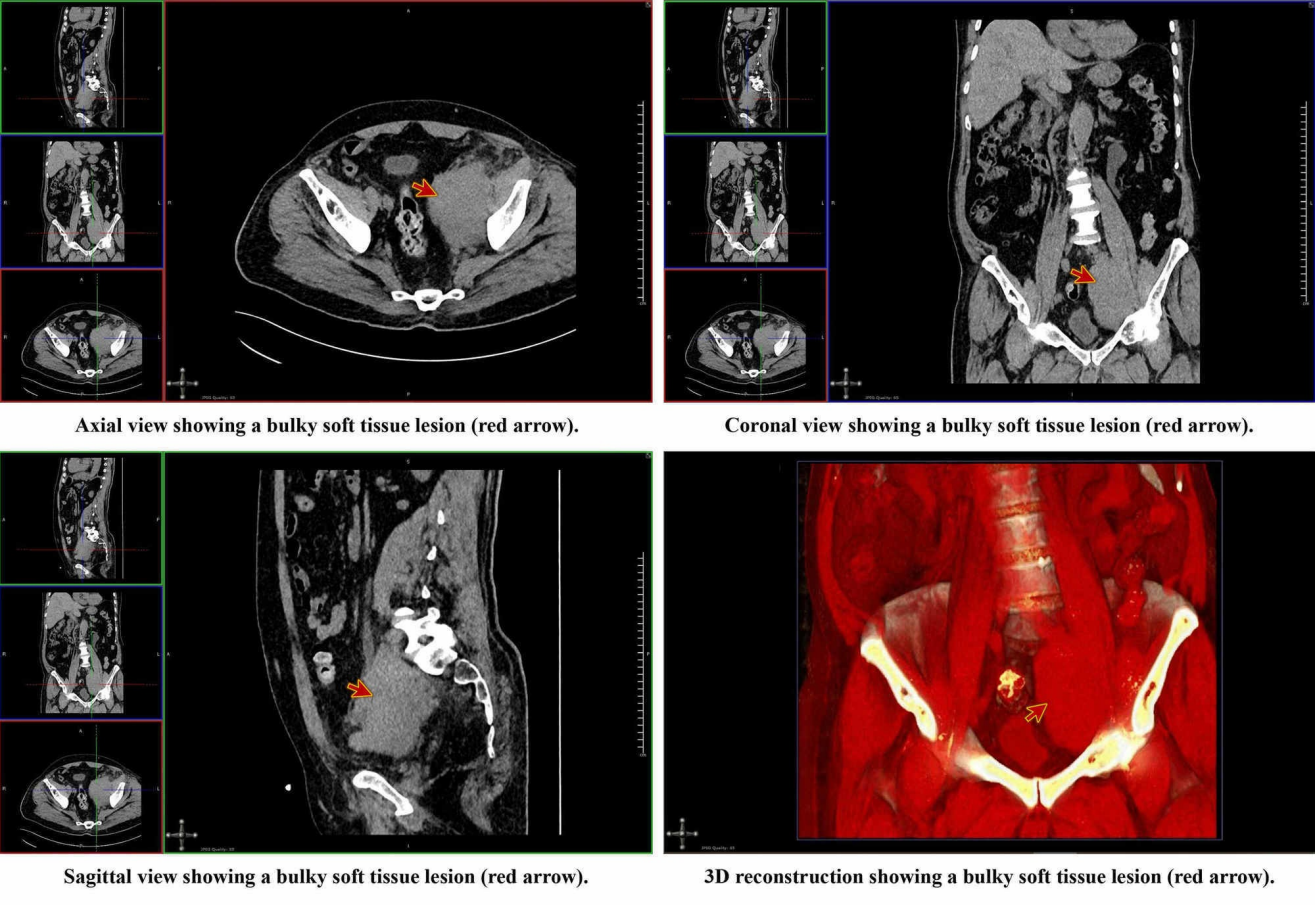
Axial, coronal, sagittal, and 3D reconstruction views of the CT of kidney, ureter, and bladder (CT KUB) CT: computed tomography

A Doppler ultrasonography was performed, which ruled out deep vein thrombosis. Laboratory tests revealed renal impairment (creatinine: 180 µmol/L, urea: 12.60 mmol/L), hypokalemia (potassium: 3.1 mmol/L), and hypochromic normocytic anemia. The liver function tests and his blood gases results were within normal limits. Bedside PoCUS revealed left kidney moderate hydronephrosis (Figure 1) and dilatation of the proximal part of the left ureter. The existence of an intra-abdominal mass that compressed both the venous system of the left leg and the left ureter was suspected. Therefore, a non-contrast CT scan of the abdomen and pelvis was indicated. It showed a bulky soft tissue lesion in the region of the distal left psoas (Figure 2) that generated an extrinsic compression of the left ureter and venous system, and multiple para-aortic lymph nodes (Video 1).

**Video 1:** CT of kidney, ureter, and bladder (CT KUB) The video shows left-sided hydronephrosis (red arrow) and hydro-ureter with bulky left peri-psoas soft tissues (blue arrow) CT: computed tomography

A whole-body fluorodeoxyglucose positron emission tomography (FDG-PET/CT) reported hypermetabolic soft tissue mass in the left psoas region highly suspicious for disease recurrence. Also, it showed suspicious malignant lesions in the lower abdomen, mesorectal regions, left iliac bone, right nasopharyngeal soft tissue, and mediastinum. CT-guided flow cytometry on left psoas muscle lesion biopsy showed approximately 47% of T cells (thymus cells) and 13% of B cells (bone marrow cells) with a partial expression of CD10. B cells were negative for the expression of the superficial light chain. However, due to the small sample provided and its low cell count and viability, the clonality was not concluded with confidence. Therefore, these results must be correlated with histopathological findings. CT-guided mass biopsy revealed atypical lymphocytic proliferation composed of small B cells expressing CD10 and CD23. These results were highly suggestive of follicular lymphoma recurrence.

Treatment

The patient was referred to the urology department, where he underwent the placement of a double-J (DJ) catheter to relieve the obstruction and ensure the flow of urine from the kidney to the bladder. Afterward, the patient was referred to the National Center for Cancer Care and Research (NCCCR) to ensure adequate disease management and to start a suitable chemotherapy plan.

Outcome and follow-up

The patient planned to have a course of six cycles of rituximab, bendamustine, and rasburicase. He received the first cycle, and then he was discharged home. He did not develop any adverse effects apart from mild nausea and vomiting. He regularly visited the out-patient department for following up. He was followed up for three months and a plain CT scan of the abdomen and pelvis was performed, which showed a significant reduction in the size of the mass.

## Discussion

The definitive diagnosis of follicular lymphoma requires the histological examination of an adequate sample of tumor tissue [4]. In the presence of superficial lymphadenopathy, excisional biopsy is the indicated method to confirm the diagnosis. In the case of deep localization lymph nodes, thick needle biopsy guided by CT or ultrasonography is the most widely used technique since it has a lower complication rate and a higher probability of achieving a final diagnosis in lymphoma cases. However, in some cases, the material obtained is of poor quality, and a surgical biopsy will be required to establish the definitive diagnosis.

Although less than 10% of patients with follicular lymphoma are found to be in stages I/II at the time of diagnosis of the disease, between 30-40% of these patients can be cured by radiotherapy (RT) of the affected field [5,6]. That is why RT has been considered the treatment of choice for the initial stages of the follicular lymphoma, with an overall survival rate of 60-80% in 10 years and a median survival of approximately 19 years [7]. On the other hand, recent research has observed that radiotherapy combined with rituximab (RTX) has achieved disease control rates and survival rates higher than those obtained by RT as a single-therapy treatment [8,9]. It is important to mention that the introduction of RTX has changed the treatment landscape of newly diagnosed patients, even those patients who are considered low risk. Disease-free survival is higher in the group of patients receiving RTX in induction and maintenance. However, the follow-up period is still too short to reach any conclusions.

In patients in advanced stages, it is recommended to start a chemotherapy regimen. Although there are a number of chemotherapy regimens with proven efficacy, no particular regimen has been established to be superior over another in terms of the overall survival rate. It is important to note that the addition of RTX to these chemotherapeutic regimens has managed to improve the results in a statistically significant way in the form of increased rates of complete remission, duration of response, and disease-free survival [8,9]. In the United States, the regimen consisting of rituximab, cyclophosphamide, hydroxydaunorubicin, vincristine, and prednisone (R-CHOP) is the induction treatment most frequently administered in patients with advanced follicular lymphoma and has become the first choice worldwide [10].

The response rate achieved with immunochemotherapy is very high; however, in most cases, the disease persists, and with rare exceptions, a more or less late relapse occurs. To avoid relapses, different therapeutic strategies have been developed, including maintenance treatments with interferon or RTX, consolidation with high-dose chemotherapy with autologous hematopoietic progenitor transplantation, and radioimmunotherapy. The treatment of relapse of follicular lymphoma depends on the efficacy achieved with the previous regimens, and schemes that do not have cross-resistance with the previous treatment should be used. In this sense, medications such as R-CHOP or bendamustine with or without RTX may be reasonable options. The NCCN clinical practice guidelines in oncology recommend treatment with an alternative line of chemoimmunotherapy [rituximab, cyclophosphamide, vincristine sulfate, and prednisone (R-CVP); fludarabine, cyclophosphamide, and rituximab (F-CR); fludarabine, cyclophosphamide, mitoxantrone, and rituximab (F-CMR); fludarabine and rituximab (FR)], radioimmunotherapy (I 131-tositumomab or Y90 ibritumomab tiuxetan) or any of the regimes of second-line used in patients with diffuse large B-cell lymphoma (DLBCL) [11].

The case we presented was an unusual case of recurrence of follicular lymphoma since the only symptom was swelling of the left leg. The availability of the bedside PoCUS was of great importance since it allowed us to discover that the patient had left hydronephrosis and left hydro-ureter.

Anatomically the ureter is divided into three parts (abdominal, iliac, and pelvic). In its iliac portion, the ureter crosses in front of the iliac vessels and the psoas muscle to enter the pelvis, where it joins the bladder. This anatomical knowledge allowed establishing a clinical correlation between the hydro-ureter, hydronephrosis, and the left leg swelling with the possible presence of an intra-abdominal compressive mass. The CT KUB confirmed the suspected diagnosis and the histopathological study of the lesion allowed to establish the diagnosis of follicular lymphoma recurrence.

Several cases of patients with either unilateral hydronephrosis or leg swelling due to a space-occupying lesion that causes compression of the venous system and ureter have been reported. These include retroperitoneal fibrosis, ureteropelvic junction obstruction, bladder distension, ureteral tumors, psoas muscle hydatid cyst, and psoas abscess [12-17].

Through this case report, we seek to emphasize the need for a thorough evaluation and the establishment of adequate clinical correlation in those patients who present with unilateral swelling of the lower extremities and hydronephrosis. Bedside PoCUS and CT are helpful to answer specific clinical questions, confirm the presence of a compressive intra-abdominal mass, and accelerate the treatment of patients.

## Conclusions

The presence of unilateral leg swelling and ipsilateral hydronephrosis increases the likelihood of the diagnosis of an intra-abdominal compressive mass suspect that compresses the ureter and venous system of the affected side. Bedside PoCUS can lead to specific clinical hypotheses and accelerate the diagnosis and treatment of patients. A previous history of follicular lymphoma should alert a high index of suspicion of possible recurrence.
